# Expression of the checkpoint kinase BUB1 is a predictor of response to cancer therapies

**DOI:** 10.1038/s41598-024-55080-y

**Published:** 2024-02-23

**Authors:** Ylenia Cicirò, Denise Ragusa, Arturo Sala

**Affiliations:** 1 Centre for Inflammation Research and Translational Medicine (CIRTM), Brunel University London, Uxbridge, UB8 3PH UK; 2 Centre for Genome Engineering and Maintenance (CenGEM), Brunel University London, Uxbridge, UB8 3PH UK

**Keywords:** Chemotherapy, Computational biology and bioinformatics

## Abstract

The identification of clinically-relevant biomarkers is of upmost importance for the management of cancer, from diagnosis to treatment choices. We performed a pan-cancer analysis of the mitotic checkpoint budding uninhibited by benzimidazole 1 gene BUB1, in the attempt to ascertain its diagnostic and prognostic values, specifically in the context of drug response. BUB1 was found to be overexpressed in the majority of cancers, and particularly elevated in clinically aggressive molecular subtypes. Its expression was correlated with clinico-phenotypic features, notably tumour staging, size, invasion, hypoxia, and stemness. In terms of prognostic value, the expression of BUB1 bore differential clinical outcomes depending on the treatment administered in TCGA cancer cohorts, suggesting sensitivity or resistance, depending on the expression levels. We also integrated in vitro drug sensitivity data from public projects based on correlation between drug efficacy and BUB1 expression to produce a list of candidate compounds with differential responses according to BUB1 levels. Gene Ontology enrichment analyses revealed that BUB1 overexpression in cancer is associated with biological processes related to mitosis and chromosome segregation machinery, reflecting the mechanisms of action of drugs with a differential effect based on BUB1 expression.

## Introduction

Despite significant improvements in clinical management of human cancers over the years, conventional therapeutic strategies alone have failed to achieve cures. According to latest statistics, cancer diagnoses have increased by 54.9% from 2020^1–3^, suggesting that more accurate biological markers should be identified and used in the clinic for efficient diagnostic and prognostic evaluations. Pan-cancer analyses of large clinical datasets focusing on gene expression represent a promising strategy for the identification of clinical biomarkers^4–6^.

Different stages of the cell cycle are tightly regulated by gateway control mechanisms known as checkpoints^7^, which are of particular interest in cancer due to its highly proliferative nature^8^. Mitotic checkpoint kinases have gained interest as therapeutic strategies in cancer, with the goal of lethal disruption of the mitotic machinery^9^. Among these, BUB1 is a highly conserved serine/threonine protein kinase involved in the mitotic checkpoint^10^. The main function of BUB1 is to organise the spindle assembly checkpoint (SAC) during mitosis, monitoring chromosome segregation and stable attachment of the spindle-microtubule polymers to kinetochores^11,12^. The SAC is composed of different proteins, such as BUB1, BUB3, MAD1, MAD2, and MAD3 (BUBR1 in higher eukaryotes), AURKB, and CDC20^11–14^. BUB1 recruits several proteins to support chromosome alignment, and its knockout in mouse models results in embryonic lethality, suggesting that it is necessary for faithful mitosis^12,15,16^. Alterations in BUB1, including deletions, point mutations, and expression changes have been observed in several malignancies^17–23^. However, due to the various biological roles of BUB1 in the cell, it is not clear how BUB1 promotes or supports tumourigenesis. Although the mechanisms of action are not fully understood and still controversial, different studies correlated BUB1 expression (and limitedly BUB1 mutations) with poor prognosis in several cancers, including breast cancer, glioma, prostate cancer, and salivary gland tumours^24–28^. Therefore, BUB1 expression might serve as a biomarker in cancer, although comprehensive analyses of BUB1 across malignancies are lacking.

Therapeutic harnessing of SAC components has been of interest in pre-clinical and clinical settings, aiming to develop increasingly effective and limitedly toxic options, such as inhibitors of AURKB, PLK1, or MPS1^9,29^. Targeting BUB1 has been shown to be a promising strategy in osteosarcoma patients, where inhibition of the kinase markedly suppressed cell proliferation, migration, invasion, and induced apoptosis; in bladder cancer, BUB1 inhibition suppressed tumour progression^30^. Previous studies have indicated that small molecule inhibitors of BUB1, such as BAY320 or BAY524, can inhibit the kinase catalytic activity in biological settings^31,32^. However, these drugs showed limited pharmacokinetic properties making them unsuitable for in vivo investigations^31,32^. On the other hand, Siemeister et al.^33^ were able to develop a new inhibitor, called BAY1816032, that is orally available, relatively non-toxic and with favourable pharmacokinetic and anti-tumour characteristics^33^. We have previously reported that BUB1 is overexpressed in adenoid cystic carcinoma^34^ and is a promising therapeutic target, as BUB1 inhibition via BAY1816032 resulted in anti-proliferative activity in both PDX and cell line models of the malignancy^35^. Other groups have shown superior combinatorial effects of BUB1 inhibition with other standard treatments both in vitro and in vivo, including taxanes and DNA damaging agents^36,37^. Therefore, single agent treatments per se might not be sufficient to trigger a considerable antitumour response, highlighting the need to identify new combinatorial strategies that can prompt anti-proliferative effects in a synergistic or additive way by modulating BUB1 levels. In pancreatic tumours, sensitivity to taxanes was also shown to be linked to an increase in BUB1 expression following the ectopic overexpression of androgen receptors^37^, indicating that BUB1 levels may dictate drug response.

Therefore, we hypothesised that BUB1 expression could be a predictor of drug responses in cancer, with the potential to extend beyond the reported relationships with taxanes and DNA damaging compounds. In this study, we performed a broad-spectrum analysis on data from The Cancer Genome Atlas (TCGA) and Genotype Tissue Expression (GTEx) consortia and in vitro drug sensitivity data from Genomics of Drug Sensitivity in Cancer (GDSC) and The Cancer Therapeutics Response Portal (CTRP) public projects to investigate the expression of BUB1 in different cancers and its role in influencing cancer response to various therapeutic strategies, with the goal to uncover BUB1 potential as biomarker in cancer.

## Materials and methods

### Data availability and sample selection

Expression and survival data for healthy and cancer tissue samples were downloaded from the TCGA and GTEx data, available in the TCGA-GTEx consortia projects from the University of California Santa Cruz (UCSC) Xena platform^38,39^. The TOIL pipeline was used to normalise gene expression of raw RNA sequencing (RNA-seq) reads from different datasets as log2(norm_count + 1). Differential expression between normal and tumour samples is presented as fold change, defined as the ratio between tumour and healthy tissues. Expression levels in counts per million (log2CPM) for detailed molecular subtypes were retrieved from the TISIDB repository^40^. Detailed clinical information on pathological features and drug data for the TCGA cohort were downloaded from the Genomic Data Commons Data Portal (available at https://portal.gdc.cancer.gov/).

### ROC

The receiver-operating characteristic (ROC) was computed in R environment using the library pROC to calculate the area under the curve (AUC) value to discern tumour vs healthy samples.

### Chi-square analysis

Chi-squared test was used to correlate clinico-pathological features and BUB1 expression. Patients were classified as “high” or “low” according to expression values relative to the mean average. Mosaic plots were constructed in R environment using the library vcd.

### Correlation of phenotypic features

Phenotypic scores for hypoxia (Ragnum Hypoxia Score, Buffa Hypoxia Score, Winter Hypoxia Score), genomic instability (fraction of genome altered, aneuploidy score, microsatellite instability MSI MANTIS, MSI sensor scores), mutational burden (mutation count) were downloaded from cBioPortal from PanCancerAtlas TCGA cancers^41,42^. Proliferation rates were downloaded as computer from Diener and Resendis Antonio^43^ (available at https://hub.docker.com/r/cdiener/proliferation). Stemness scores (mRNAsi and mDNAsi) were retrieved as computed by Malta et al.^44^. Correlations between expression values and scores were calculated by Pearson correlation coefficients and deemed statistically significant by *p* values < 0.05 and Pearson value ≥|0.2|.

### Survival analysis

Survival data (overall survival; OS) for the TCGA cohort was downloaded from the TCGA and GTEx data, available in the TCGA- GTEx consortia projects from the University of California Santa Cruz (UCSC) Xena platform^39^. Survival probability was determined by Kaplan–Meier plots generated using R libraries survminer (0.4.9) and survival (3.2–11). Patients were classified as “high” and “low” by mean average expression cut-offs.

### Differential expression analysis

Expression data in the form of htseq counts for all genes were downloaded from GDC TCGA cohort for each cancer from the University of California Santa Cruz (UCSC) Xena platform^39^. limma (version 3.48.3) in R studio was used to perform differential gene expression analysis between “high” and “low” BUB1 expression groups by mean average expression cut-offs. The statistically significant genes were filtered by a false discovery rate (FDR) value of < 0.01.

### Gene set enrichment analysis (GSEA)

Gene signatures for drug responses were retrieved from the GEO Signatures of Differentially Expressed Genes for Small Molecules^45,46^. Custom gene sets were run on htseq count expression values of “high” and “low” BUB1 expression groups using the GSEA software v4.2.3. 1000 permutations were performed with the weighted Signal2Noise metric to compute Normalized Enrichment Scores (NES).

### Drug sensitivity correlations

Drug sensitivity (IC50 values) and BUB1 expression data (in RMA values) for cell lines of the Genomics of Drug Sensitivity in Cancer (GDSC) project were downloaded from GDSC1000 resources (available at https://www.cancerrxgene.org/gdsc1000/GDSC1000_WebResources/Home.html). Data from the Cancer Therapeutics Response Portal (CTRP) v2 was retrieved from the NCI CTD2 Data Portal (available at https://ctd2-data.nci.nih.gov/Public/Broad/) to extract drug sensitivity (AUC response curve values) and BUB1 expression data (average log2 gene expression values). Correlations were calculated by Pearson correlation coefficients and deemed statistically significant by *p* values < 0.05 and Pearson value  ≥|0.2|.

### Drug set enrichment analysis

Drug set enrichment analysis was performed on the Drugmonizome online platform^47^ by inputting drug names to retrieve enrichments terms in the Drug Repurposing Hub of Mechanisms of Action. Significant terms were selected by FDR < 0.05.

### Gene ontology analysis

Gene Ontology (GO) enrichment analysis was performed on ExpressAnalyst (available at www.expressanalyst.ca) using the Gene Ontology Biological Process (GO:BP) and Gene Ontology Cellular Component (GO:CC) databases. Statistical significance was determined using *p* value < 0.05.

### Data analysis and statistical tests

Plots were generated in Microsoft Excel 365 or in R studio 4.3.0 using the libraries ggplot2 (v 3.4.2) and heatmap3 (v 1.1.9). Venn diagrams were generated on EVenn^48^. Statistical significance was calculated by in R environment (stats v 4.3.0 and ggpubr v 0.6.0). A *p* value ≤ 0.05 was considered statistically significant, and *p* values = 0.1 was considered weakly significant. Statistical significance is represented as: *p* value ≤ 0.05 (*), 0.001 (**), 0.0001 (***), and 0.00001 (****).

## Results

### BUB1 is overexpressed in cancer and particularly elevated in aggressive molecular subtypes

**Figure 1:**
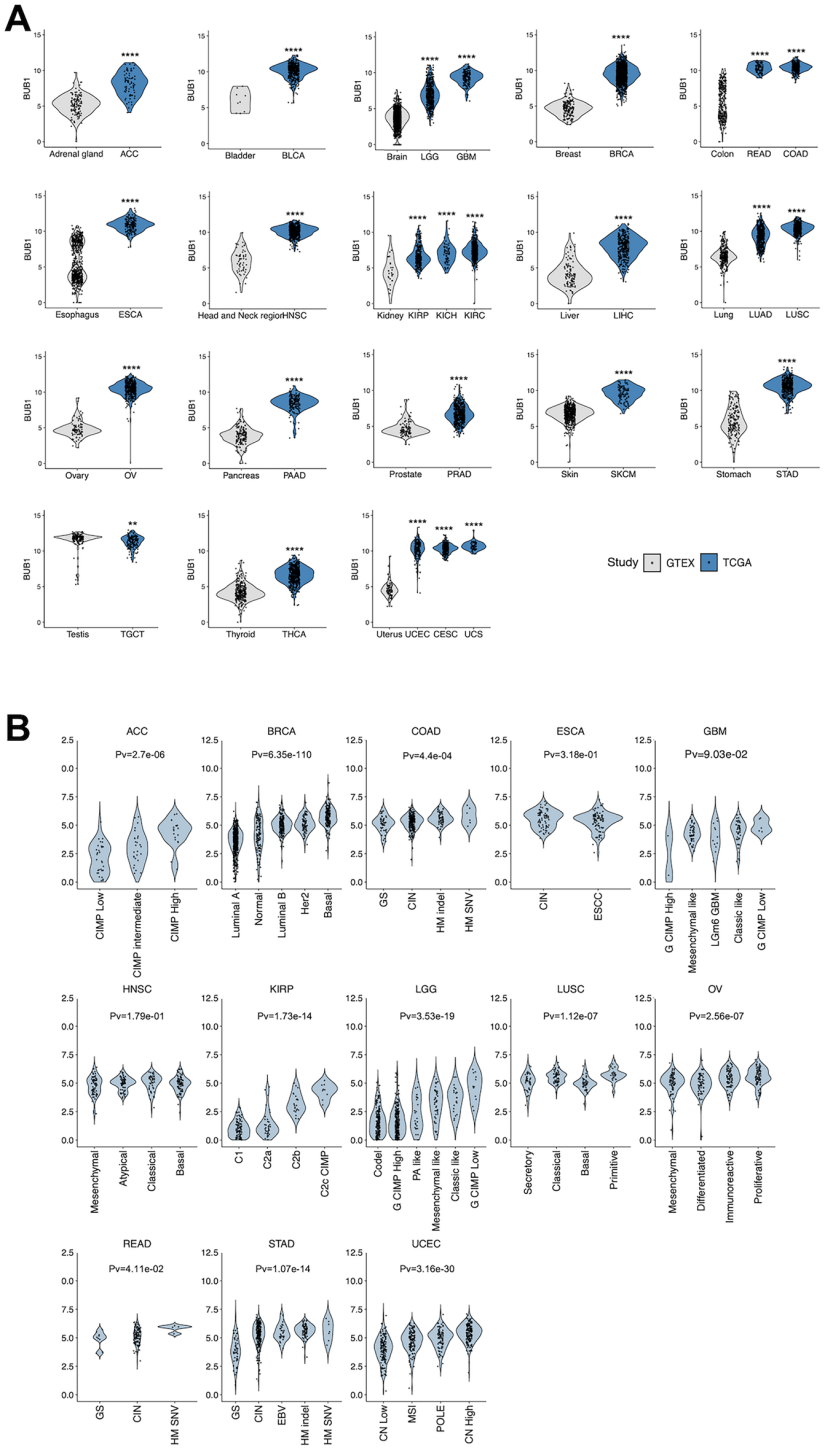
Expression of BUB1 in different cancers of the TCGA project. (**A**) BUB1 expression levels in tumour sites (TCGA) compared to non-matching healthy tissues (GTEx). Statistical significance was calculated by Wilcoxon’s test. (**B**) BUB1 expression in different molecular subtypes by cancer site. Differences in mean expression across groups was calculated by Kruskal–Wallis test (Pv = *p* value).

We interrogated the expression of the BUB1 gene in 25 cancers of the TCGA cohort compared to unmatched healthy tissues from the GTEx database (Supplementary File 1) and observed a widespread dysregulation of BUB1 in all cancers analysed (Fig. 1A). By calculating the average fold change between tumour expression and normal tissue expression, most cancers presented an upregulation of the gene, with only one instance of downregulation in TGCT (Supplementary Fig. 1A). In particular, the highest fold changes were observed in gynaecological tumours (UCS, CESC, UCEC, and OV), GBM, and tumours of the gastrointestinal tract (ESCA, COAD, READ, and STAD). Based on the significant differential expressions, we conducted a ROC analysis to determine the discriminative power of BUB1, in light of a potential use as a diagnostic marker. The expression of BUB1 showed a strong performance in distinguishing normal vs tumour tissues in the majority of sites, as determined by AUC values defining sensitivity and specificity of the marker (Supplementary Fig. 1B). The highest AUC values closest to 1.0 (i.e. with the highest sensitivity and specificity) were achieved by UCS, CESC, GBM, BRCA, READ, and OV, with most cancers averaging above 0.8, indicating a potential diagnostic use of BUB1. Moreover, a detailed analysis of BUB1 expression in different tumours revealed distinct expression levels by molecular subtype (Fig. 1B). A higher expression of BUB1 was observed in CpG island methylator phenotype (CIMP) in ACC and KIRP. In brain tumours (GBM and LGG), BUB1 expression was elevated in the G-CIMP low subtype. BUB1 expression was also distinct in BRCA subtypes, with the highest expression in basal BRCA. In gastrointestinal cancers (COAD, STAD, and READ), hypermutated with elevated single nucleotide variants (HM-SNV) tumours had the highest level of the gene. Highly proliferative subtypes of LUSC (“primitive”) and OV (“proliferative”) also showed high BUB1 expressions. Overall, these results indicated that BUB1 is overexpressed in the majority of TCGA tumours and that its expression is elevated in molecular subtypes associated with clinical aggressiveness.

### BUB1 expression associates with aggressive clinical and phenotypic features

**Figure 2:**
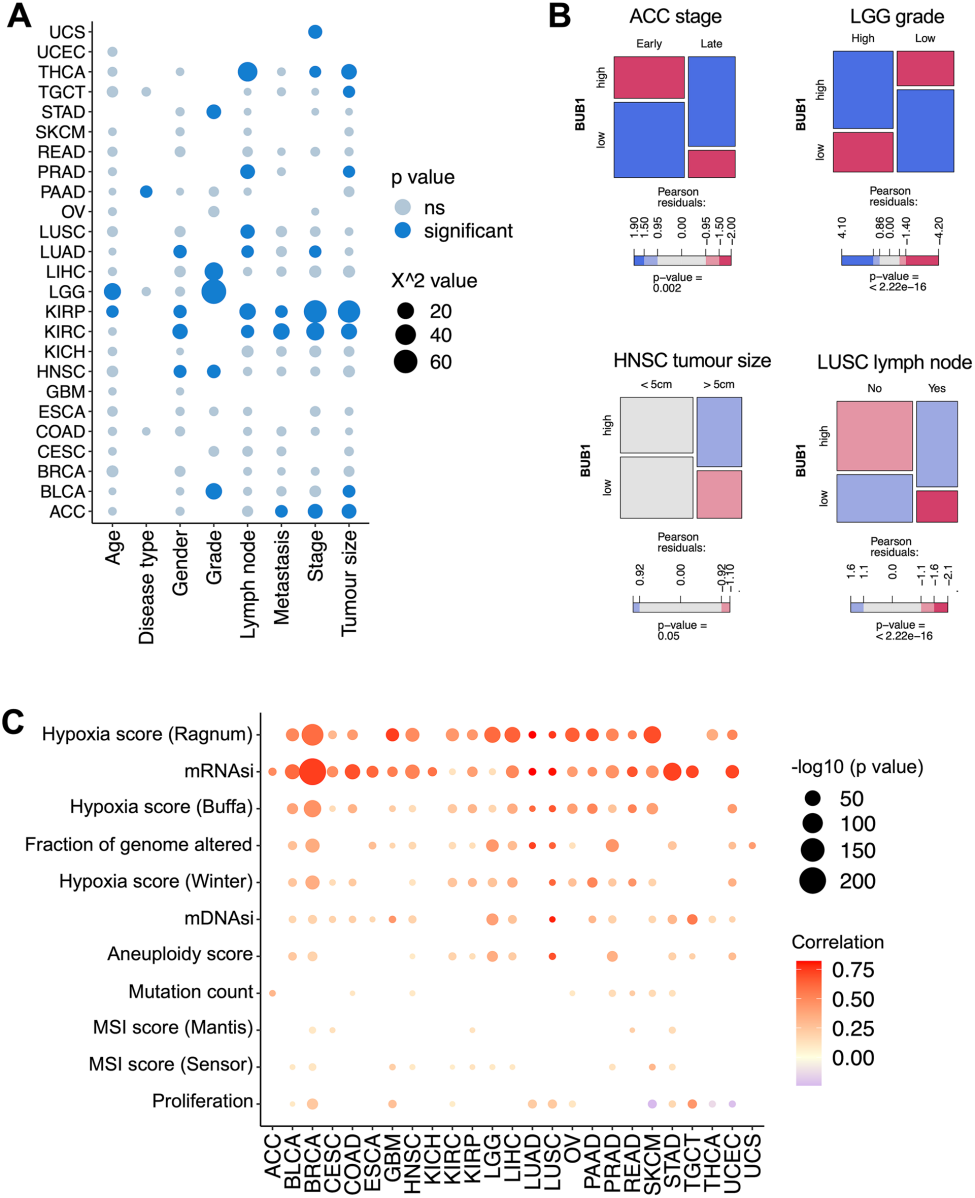
Clinical and phenotypical features associates with BUB1 expression levels in TCGA cancers. (**A**) Chi-squared analysis of correlation between BUB1 levels and clinico-pathological features. Dots represent X^2 values and statistical significance  < 0.05 is marked in blue. (**B**) Representative mosaic plots of significant Chi-squared associations showing the proportion of “high” and “low” groups in the respective clinical category. (**C**) Pearson correlation analysis between BUB1 expression and phenotypic features of hypoxia (Ragnum Hypoxia Score, Buffa Hypoxia Score, and Winter Hypoxia Score); stemness (mRNAsi and mDNAsi scores); genomic instability (fraction of genome altered, aneuploidy score, microsatellite instability MSI MANTIS, and MSIsensor scores); mutational burden (mutation count); and proliferation score. Size of the dots represent the statistical significance in −log10 (*p* value). The colour of the dots indicates the strength of the correlation by Pearson coefficient.

On the basis of BUB1’s association with aggressive cancer subtypes (Fig. 1B), we explored the association between BUB1 expression and clinico-pathological features by Chi-square analysis (Fig. 2A,B, Supplementary Table 1). Specifically, we found statistically significant associations between the expression of BUB1 and TNM classifiers (i.e. tumour size, lymph node, and metastasis status), grading and staging in several tumours (Fig. 2A). A high expression of BUB1 was associated with advance disease status in ACC, BLCA, HNSC, KIRC, KIRP, LGG, LIHC, LUAD, LUSC, PRAD, STAD, and THCA (Fig. 2A). As exemplified in Fig. 2B, advanced tumour grading (“high grade”) in LGG and late staging in ACC are associated with high BUB1 levels; similarly, lymph node invasion in LUSC and larger HNSC tumours over 5 cm in size present a higher proportion of high BUB1 expressors.

Next, we expanded the associations to phenotypic features associated with tumour aggressiveness, namely hypoxia (Ragnum Hypoxia Score, Buffa Hypoxia Score, and Winter Hypoxia Score); stemness (mRNAsi and mDNAsi scores); genomic instability (fraction of genome altered, aneuploidy score, microsatellite instability MSI MANTIS, and MSIsensor scores); mutational burden (mutation count); and proliferation score^43^ (Fig. 2C). Strongest correlations were observed with hypoxia and stemness scores, as determined by Pearson correlation coefficients and statistical power by *p* value (Fig. 2C). Hypoxia scores were significantly correlated with BUB1 expression in 19 cancers out of 25, with a median Pearson correlation of 0.41 (range 0.12–0.82); stemness scores were correlated in 24 cancers with a median of 0.47 (range 0.1–0.83). While several tumours showed statistically significant correlations with genomic instability in the form of fraction of genome altered (16 cancers out of 25, median = 0.28, range 0.13–0.73) and aneuploidy (12 cancers out of 25, median = 0.21, range 0.1–0.69), MSI and mutation counts presented a weaker correlation in fewer cancers [13 cancers out of 25 for MSI, median = 0.1 (range 0.08–0.31); 8 cancers out of 25 for mutation counts, median = 0.1 (range 0.09–0.31)]. Proliferative scores showed a modest association with BUB1 expression (12 cancers out of 25, median = 0.22, range 0.08–0.44).

### BUB1 expression has prognostic value according to chemotherapy treatment

**Figure 3:**
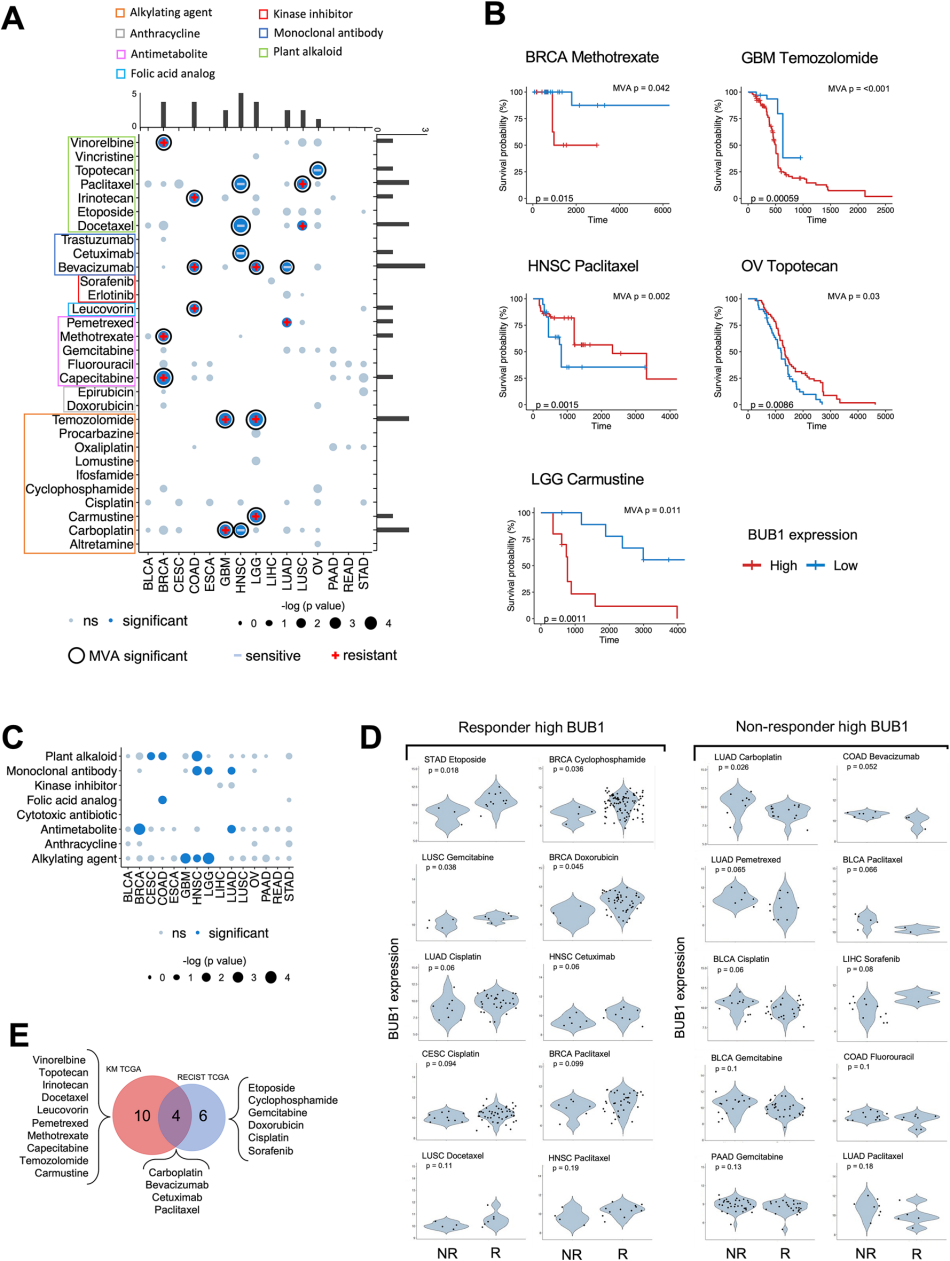
Prognostic value of BUB1 by treatment type in different cancers from the TCGA database. (**A**) Summary of results of Kaplan–Meier analysis by overall survival (OS) according to BUB1 expression levels (high-low) by drug administered. Dark blue dots indicate significant association by *p* value (< 0.05). The size of the dots indicates the significance in −log10 (*p* value). Black circles signify a significant result (*p* value < 0.05) by multivariant analysis (MVA). + symbol indicates “resistant” (Kaplan–Meier result i.e. high expression of BUB1 is associated with a worse outcome for the given drug), and – indicates “sensitive” when a high expression of BUB1 results in a favourable OS. External bar charts show the sum of the significant associations by drug (horizontal) and by cancer (vertical). Drugs are grouped by classes which are indicated by the coloured boxes around the drug names. (**B**) Representative Kaplan–Meier plots of significant OS changes between BUB1 high and BUB1 low cohorts by drug and by cancer site. MVA = *p* value of multivariate analysis, p = *p* value of Kaplan–Meier analysis. (**C**) Summary of results of Kaplan–Meier analysis by overall survival (OS) according to BUB1 expression levels (high-low) by drug class. Dark blue dots indicate significant association by *p* value (< 0.05). The size of the dots indicates the significance in −log 10 (*p* value). (**D**) Differential expression of BUB1 by drug between responders (R) and non-responders (NR) according to the RECIST classification of disease progression. On the left, cancer types and drugs where the expression of BUB1 was higher in the responders (“sensitive”); on the right, cases where the expression of BUB1 was higher in non-responders (“resistant”). (**E**) Venn diagram showing the intersect between statistically significant drugs identified by the TCGA Kaplan–Meier analysis (KM) and the RECIST data. NR = non responder; R = responder.

Given the differential expression of BUB1 in distinct cancer subtypes associated with aggressiveness, we sought to determine the prognostic relevance of BUB1 expression. First, we performed Kaplan–Meier analyses using expression levels of BUB1 (“high” or “low” based on mean cut-offs) by cancer site and found that a high expression of BUB1 resulted in worse overall survival (OS) in LUAD, UCEC, PAAD, SKCM, LIHC, ACC, KIRC, KIRP, and LGG (Supplementary Fig. 2). Next, we asked whether BUB1 levels could be associated with clinical outcomes based on the treatment administered, by leveraging clinical data available from TCGA (Supplementary File 1). Kaplan–Meier analyses subset by drug revealed significant associations with OS in several cancer sites, which were further validated by multivariate analysis (MVA) to assess BUB1 expression as an independent variable (Fig. 3A; Supplementary Fig. 3). We classified the Kaplan–Meier results as “resistant” when a high expression of BUB1 is associated with a worse outcome for the given drug, and “sensitive” when a high expression of BUB1 results in a favourable OS, as exemplified by representative Kaplan–Meier plots in Fig. 3B (Supplementary Fig. 4). The most significant associations were found for drugs belonging to plant alkaloids (vinorelbine, topotecan, paclitaxel, irinotecan, and docetaxel) and monoclonal antibodies (cetuximab and bevacizumab), with the most frequent cancer types being HNSC, BRCA, COAD, and LGG (Fig. 3A). By analysing the OS of patients classified by drug class (as shown by squares in Fig. 3A) to improve statistical power of low sample numbers (Supplementary File 1), we further corroborated the association of BUB1 with plant alkaloids, monoclonal antibodies, and alkylating agents (Fig. 3C; Supplementary Fig. 5). These results showed that BUB1 expression bears differential prognostic values for OS by drug and by cancer type (Fig. 3A,B).

In order to expand the scope of the prognostic power of BUB1 beyond the OS metric, we extracted drug response data of TCGA patients^39^ according to the RECIST classification system of disease progression. We interrogated the expression of BUB1 in responsive cohorts (complete response + partial response) against non-responsive cohorts (stable disease + progressive disease) to identify whether expression levels of BUB1 are different according to the response to the drug (Fig. 3D; Supplementary Fig. 6). Similarly to the Kaplan–Meier results (Fig. 3A), associations were classified as “resistant” when BUB1 expression was higher in the non-responsive group, or “sensitive” if BUB1 was higher in the responsive class (Fig. 3D; Supplementary Fig. 6). We identified significant associations of sensitivity for etoposide (STAD), cyclophosphamide and doxorubicin (BRCA); resistant drugs included carboplatin (LUAD) and bevacizumab (COAD). Gemcitabine, cisplatin and paclitaxel showed cancer-specific responses (Fig. 3D). Together with the Kaplan–Meier analysis on OS (Fig. 3A,B), we identified a total of 20 drugs with differential clinical outcomes based on BUB1 expression (Fig. 3E), with an overlap of 4 drugs between the Kaplan–Meier and RECIST analysis (i.e. carboplatin, bevacizumab, cetuximab, and paclitaxel). Drug enrichment analysis revealed that the mechanisms of action of the 20 drugs are primarily DNA alkylating agents, topoisomerase inhibitors, or thymidylate synthase inhibitors (Supplementary Fig. 7). Overall, these results indicate that BUB1 expression is associated with clinical outcomes according to the drug administered, with cancer-specific effects of drugs preferentially involved in DNA-related processes.

### Prediction of drugs affected by BUB1 expression levels

**Figure 4:**
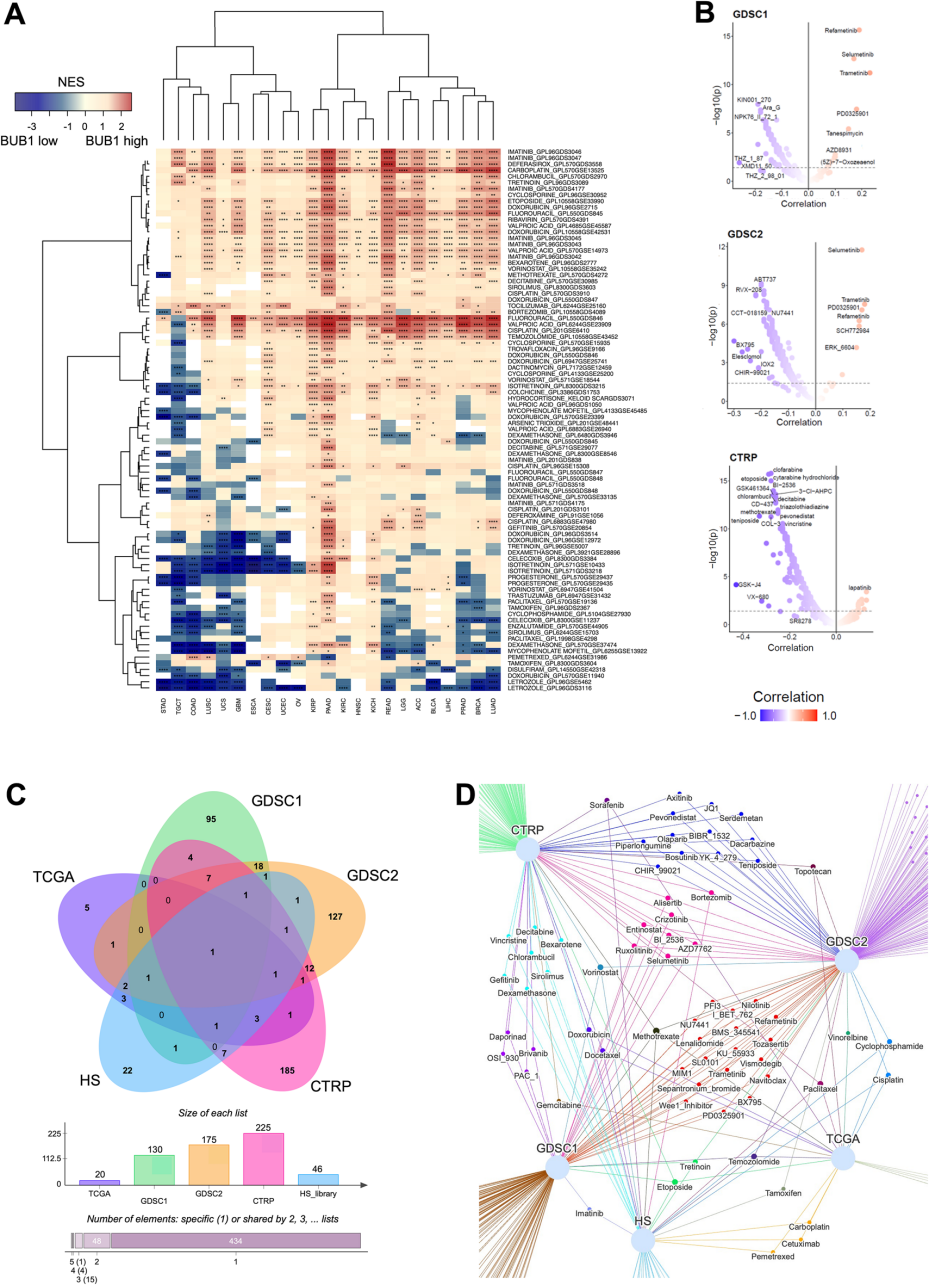
Drug response prediction based on BUB1 expression. (**A**) Heatmap representing GSEA enrichment values (NES) for drug response signatures by cancer type in BUB1 high and BUB1 low cohorts of the TCGA project. FDR values ≤ 0.05 (*), 0.001 (**), 0.0001 (***), and 0.00001 (****). (**B**) Volcano plots of correlations between drug sensitivity and BUB1 expression in cell lines of the GDSC1, GDSC2, and CTRP repositories. Correlation is calculated as Pearson coefficient and is represented by the colour gradient and plotted against −log10 (*p* value). (**C**) Venn diagram representing the intersects among statistically significant drugs associated with BUB1 expression identified through the different analyses (TCGA, GDSC1, GDSC2, CTRP, and HS). HS = Homo sapiens geneset library from the GEO Signatures of Differentially Expressed Genes for Small Molecules used for GSEA in (**A**). The bar chart underneath shows the sizes of each drug list and the number of common intersects by groups. (**D**) Venn network displaying common drugs shared among the lists obtained by analysis of TCGA, GDSC1, GDSC2, CTRP, and HS.

Based on the associations of BUB1 expression with clinical outcomes of specific drugs, we asked whether patient cohorts with high BUB1 or low BUB1 have distinct gene expression patterns associated with response to drugs. We extracted a panel of gene signatures associated with drug response from the GEO Signatures of Differentially Expressed Genes for Small Molecules^46^ and performed GSEA to identify gene sets that are differentially enriched between the BUB1 high and BUB1 low groups from the TCGA cohort (Fig. 4A; Supplementary File 2). The heatmap in Fig. 4A shows GSEA enrichment values (NES) for drug response signatures by cancer type, identifying differential responses by compound; positive NES values (i.e. enriched in the BUB1 high group) suggest that the responsive genes to the given drug are expressed in patients with a high expression of BUB1, while negative NES values indicate an enrichment in the BUB1 low group (hence, high BUB1 expression does not express drug responsive genes). With this approach, 46 statistically significant compounds were retrieved with a differential enrichment depending on the level of BUB1 (Supplementary File 2).

In order to extend the identification of candidate drugs that are influenced by BUB1 expression, we mined data from the GDSC and CTRP repositories of in vitro drug screens in various cell lines^49–52^. We performed a correlation analysis between drug response (measured in IC50 in GDSC1-2 or AUC for CTRP) and BUB1 expression to identify potential compounds with distinct efficacy based on the level of the gene (Fig. 4B). We found 130, 175, and 225 compounds for GDSC1, GDSC2, and CTRP, respectively, with a Pearson correlation value of  ≥|0.1| and *p* value below the threshold of 0.05 (Supplementary File 3). The majority of correlations were negative values, indicating that higher expression of BUB1 inversely affects the efficacy of the drug (i.e. at high BUB1 levels, the drug is more effective with a lower IC50/AUC).

To streamline the identified drugs, we overlapped the significant results from the analyses on the clinical outcomes of the TCGA cohort (Fig. 3), the GSEA enrichments (Fig. 4A), and the in vitro predictions of the GDSC and CTRP repositories (Fig. 4B), as shown in Fig. 4C. We identified 1 drug (methotrexate) in common to all analyses, 4 drugs (paclitaxel, temozolomide, vorinostat, and etoposide) common to at least 4 groups, and 15 drugs common to at least 3 groups (Fig. 4C,D). A focused list of shared drug hits, their mechanisms of action, and their previous reports is compiled in Table 1, comprising of compounds with significant associations with BUB1 with the highest number of overlaps between analyses and prioritising compounds with a clinical association with the TCGA cohorts. The full list of candidate compounds with differential responses (by various metrics) according to BUB1 expression levels can be retrieved in Supplementary File 3.

**Table 1:** Focused list of drugs associated with BUB1 expression.

Drug	Drug class	Target	Overlap	Analysis	Clinical/in vitro	Resistant	Sensitive	References
Methotrexate	Antimetabolite	DHFR	5	TCGA|GDSC1|GDSC2|CTRP|HS_library	Clinical + in vitro	BRCA	in vitro	^53^
Paclitaxel	Taxane	Microtubules	4	TCGA|GDSC2|CTRP|HS_library	Clinical + in vitro	LUSC, BLCA, LUAD	HNSC, in vitro	^36^
Etoposide	Topoisomerase inhibitor	TOP2A	4	TCGA|GDSC1|CTRP|HS_library	Clinical + in vitro		STAD, in vitro	^53^
Temozolomide	Alkylating agent	DNA crosslink	4	TCGA|GDSC1|GDSC2|HS_library	Clinical + in vitro	LGG, GBM	in vitro	^53,54^
Vorinostat	HDAC inhibitor	HDAC	4	GDSC1|GDSC2|CTRP|HS_library	in vitro		in vitro	This study
Docetaxel	Taxane	Microtubules	3	TCGA|CTRP|HS_library	Clinical + in vitro	LUSC	HNSC, in vitro	^36^
Cisplatin	Alkylating agent	DNA crosslink	3	TCGA|GDSC2|HS_library	Clinical + in vitro	BLCA, in vitro	LUAD, CESC	^36^
Doxorubicin	Anthracycline	TOP2A, DNA intercalation	3	TCGA|CTRP|HS_library	Clinical + in vitro		BRCA, in vitro	^53^
Topotecan	Topoisomerase inhibitor	TOP2A	3	TCGA|GDSC2|CTRP	Clinical + in vitro		OV, in vitro	^53^
Cyclophosphamide	Alkylating agent	DNA crosslink	3	TCGA|GDSC2|HS_library	Clinical + in vitro		BLCA, in vitro	This study
Alisertib	Aurora A inhibitor	Aurora A	3	GDSC1|GDSC2|CTRP	in vitro		in vitro	This study
AZD7762	Checkpoint kinase inhibitor	Chk	3	GDSC1|GDSC2|CTRP	in vitro		in vitro	This study
Entinostat	HDAC inhibitor	HDAC	3	GDSC1|GDSC2|CTRP	in vitro		in vitro	This study
Ruxolitinib	JAK Inhibitors	JAK1, JAK2	3	GDSC1|GDSC2|CTRP	in vitro		in vitro	This study
BI_2536	PLK1 inhibitor	PLK	3	GDSC1|GDSC2|CTRP	in vitro		in vitro	This study
Bortezomib	Proteasome inhibitor	26S proteasome	3	GDSC2|CTRP|HS_library	in vitro		in vitro	This study
Tretinoin	Retinoid (differentiating agent)	RAR	3	GDSC1|GDSC2|HS_library	in vitro		in vitro	This study
Sorafenib	Tyrosine kinase inhibitor	PDGFR, KIT, VEGFR, RAF	3	TCGA|CTRP|HS_library	Clinical + in vitro		LIHC, in vitro	This study
Crizotinib	Tyrosine kinase inhibitor	ALK, HGFR, c-MET, ROS1, RON	3	GDSC1|GDSC2|CTRP	in vitro		in vitro	This study
Selumetinib	Tyrosine kinase inhibitor	MEK1, MEK2	3	GDSC1|GDSC2|CTRP	in vitro	in vitro		This study
Gemcitabine	Antimetabolite	CMPK1, RRM1, TYMS	2	TCGA|CTRP	Clinical + in vitro	BLCA, PAAD	LUSC, in vitro	^36^
Carboplatin	Alkylating agent	DNA crosslink	2	TCGA|HS_library	Clinical + in vitro	GBM, LUAD	HNSC	^36,55^
Vinorelbine	Alkylating agent	Microtubules	2	TCGA|GDSC2	Clinical + in vitro	BRCA	in vitro	This study
Pemetrexed	Antimetabolite	TYMS, GARFT	2	TCGA|HS_library	Clinical + in vitro	LUAD		This study
Cetuximab	Monoclonal antibody	EGFR	2	TCGA|HS_library	Clinical + in vitro	COAD, LGG	LUAD, HNSC	This study
Irinotecan	Topoisomerase inhibitor	TOP1	1	TCGA	Clinical	COAD		^36^
Carmustine	Alkylating agent	DNA crosslink	1	TCGA	Clinical	LGG		This study
Capecitabine	Antimetabolite	TYMS	1	TCGA	Clinical	BRCA		This study
Leucovorin	Antimetabolite	5FdUMP	1	TCGA	Clinical	COAD		This study
Bevacizumab	Monoclonal antibody	VEGF	1	TCGA	Clinical	COAD, LGG	LUAD	This study

### Biological relevance of drugs associated with BUB1 expression

**Figure 5:**
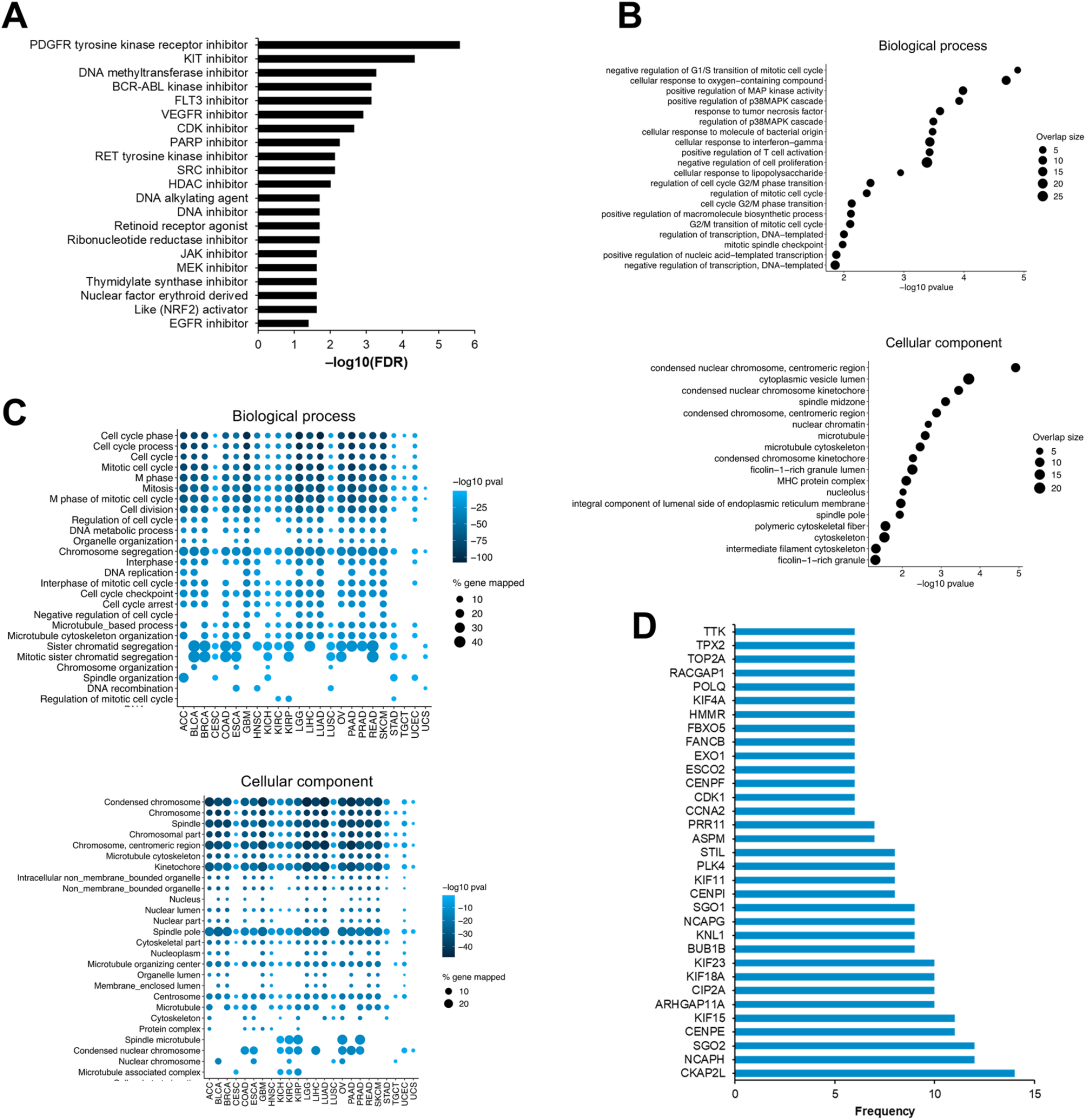
Biological relevance of drugs associated with BUB1 expression and BUB1 high vs BUB1 low TCGA cohorts. (**A**) Drug set enrichment analysis showing significant terms associated with mechanisms of action of all the identified drugs. (**B**) Drug set enrichment analysis on biological and cellular components based on Gene Ontology (GO) analysis on gene expression profiles of the queried drugs. The size of the dots represents the proportion of overlapped drugs for each GO category. (**C**) Top 25 biological processes (top) and cellular component (bottom) enriched in differentially expressed genes in patients with high vs low expression of BUB1 by cancer site. The size of the dots represents the percentage of genes mapped and the colour grading indicates the strength of the statistical significance expressed in −log10 (*p* value). (**D**) Top differentially expressed genes by occurrence in all cancers analysed.

Next, we sought to uncover the biological relevance of the BUB1-drug associations. Drug set enrichment analysis on all statistically significant candidate drugs (Supplementary File 3) revealed common mechanisms of action shared by the compounds (Fig. 5A), with an enrichment in cellular receptor inhibitors (PDGFR tyrosine kinase receptor, KIT, BCR-ABL, FLT3, VEGFR), PARP inhibitors, and DNA alkylating agents (Fig. 5A). We also looked in detail at the targets of the top 20 hits from the GDSC1/2 and CTRP analysis, as well as all significant compounds identified in the TCGA analyses and GSEA of the HS library (Supplementary File 4) and found that a large number of drugs targeting the MEK/ERK pathway, growth factor receptors (PDGFR, KIT, VEGFR), and components of the cell cycle (Cdk, PLK1). Drug set enrichment analysis also revealed common biological and cellular components shared by the compounds based on Gene Ontology analysis on gene expression profiles of the queried drugs (Fig. 5B). The top enriched biological processes belonged to chromosome structure (condensed nuclear chromosome centromeric region, kinetochore, spindle midzone, microtubule) and cytoplasmic transport (cytoplasmic vescicle lumen). Accordingly, molecular functions were enriched for cell cycle functions (cell cycle regulation, G2/M phase transition, mitosis, MAPK pathway, spindle checkpoint, cytoskeleton) and cellular metabolism and transport (response to polysaccharides, oxygen-containing compounds) (Fig. 5B).

Finally, we performed a differential expression analysis between patients with high levels of BUB1 versus low levels of BUB1 for each cancer site of the TCGA cohort. GO analysis of the differentially expressed genes revealed enrichments in cell cycle regulation, mitotic processes, chromosome structure, cytoskeleton and microtubule assembly, and vescicular transport (Fig. 5C), akin to the enriched processes of BUB1-correlated drug responses (Fig. 5B), with most cancer sites showing similar patterns of GO terms. By extracting the top 30 differentially expressed genes by FDR for each cancer, we identified the most frequently occurring genes (Fig. 5D), which are known to participate in cell cycle regulation, particularly chromosome segregation. The most frequent genes were CKAP2L (Cytoskeleton Associated Protein 2 Like), NCAPH (Non-SMC Condensin I Complex Subunit H), and SGO2 (Shugoshin 2), which were the top differential genes in more than 50% of cancers. Additional recurring genes were members of the kinesin superfamily proteins (KIFs), centromeric proteins (CENP), cyclins (CDK), and members of kinetochore/microtubule maintenance (e.g. ARHGAP11A, KNL1, STIL, ASPM) (Fig. 5D). In summary, these results suggest that BUB1 overexpression in cancer is associated with biological processes related to mitosis and chromosome segregation machinery, as well as cellular signalling and transport, which reflect the mechanisms of action and response of drugs with a differential effect based on BUB1 expression.

## Discussion

Despite significant advances in therapeutic options for cancer treatment, tackling disparities in clinical outcomes among patients remains a major clinical challenge. Considerable research efforts have been dedicated to identify and harness treatment response biomarkers with the promise of improving disease management. Here, we explored the potential of the mitotic checkpoint kinase BUB1 expression as a cancer biomarker and particularly its association with drug responses, stemming from previous studies on BUB1 dysregulation in cancer and its potential exploitation for therapeutic purposes^34–37^. We uncovered several drugs that may be influenced by the expression of BUB1 and sought to explore the biological significance behind the associations with specific drug classes.

Given BUB1’s role in establishing the mitotic spindle checkpoint to ensure chromosome segregation fidelity, it was unsurprising to find drugs affecting microtubule stabilisation and/or dynamics (e.g. docetaxel, paclitaxel, vinorelbine). In fact, the majority of differentially expressed genes between BUB1 high and BUB1 low patients in our analysis belonged to cell cycle regulation and specifically to the mitotic machinery. It has been previously reported that pharmacological inhibition of BUB1 has synergistic activity with the microtubule-stabilising taxanes paclitaxel and docetaxel, and DNA-repair-related ATR and PARP inhibitors in vitro^36^. In vivo, the combination of BUB1 inhibition with paclitaxel or with PARP inhibitors superseded single drug treatments^36^, also highlighting the potential of exploiting BUB1 modulation to achieve improved drug response and minimise resistance. We identified cancer-specific responses to paclitaxel and docetaxel in our analysis; most tumours showed worse clinical outcomes at high levels of BUB1 and concomitant expression of taxane response signatures in BUB1 low cohorts, indicating resistance; however, in vitro drug screens negatively correlated with efficacy. Mechanisms of response and resistance to taxanes, which can largely vary across cancer types and between patients, include metabolic changes, microtubule expression, tumour-specific signalling pathways, and androgen receptor status^56^. For instance, ectopically expressed androgen receptors reduced sensitivity to taxanes (docetaxel and cabazitaxel) in prostate cancer cell lines, which could be reverted by disruption of BUB1^37^. Resistance to taxanes is also linked to high chromosomal instability^56,57^^,^^58^ which also reflects our observations of paclitaxel and docetaxel resistance in LUSC, BLCA, and LUAD, which presented among the highest correlations with aneuploidy and genomic alterations scores associated with BUB1 expression.

We also matched previously reported associations of BUB1 with alkylating agents^36^. Our results showed sensitivity to cisplatin and carboplatin in most cancers with high BUB1. Accordingly, BUB1 inhibition was antagonistic to cisplatin^36^, and cisplatin exposure was found to increase BUB1 expression by computational screens^53^. Temozolomide was a high-confidence hit in our findings, with in vitro screens and gene expression analyses indicating sensitivity with high BUB1 levels. Brain tumours GBM and LGG, however, appeared to be unresponsive to DNA crosslinkers with high BUB1. Platinum resistance in brain tumours is a major clinical challenge, often driven by tumour heterogeneity and microenvironmental cues such as hypoxia^59^. In fact, elevated BUB1 expression correlated with clinically aggressive LGG and GBM subtypes (e.g. G-CIMP associated with worse outcomes^60^), as well as hypoxia scores. In vitro, temozolomide was found to have a neglectable additive effect when in conjunction with BUB1 inhibition in glioblastoma^54^, suggesting that, while BUB1 might not be therapeutically useful in these tumours, its expression could aid in risk stratification for predicting efficacy to alkylating compounds.

We found several significant associations with antimetabolites, including previously unidentified resistance to thymidylate synthase/folate analogue drugs (e.g. pemetrexed, capecitabine, leucovorin) with high BUB1 expression. Methotrexate was found to be in common to all analysis performed, with a BUB1-high-associated sensitive profile by cell line and gene expression profiles, but an adverse outcome in BRCA. While not reported in cancer, an in silico drug screening analysis identified methotrexate to inhibit BUB1 expression in the context of birth defects^61^. Antagonistic activity between gemcitabine and 5-fluorouracil with BUB1 inhibitors has also been reported^36^. The biological association of these drugs to BUB1’s activity may be related to its involvement in DNA repair, as demonstrated by the requirement of BUB1 phosphorylation by ATM following radiation-induced genotoxicity^62^. Accordingly, combinatorial treatment with ATR inhibitors showed synergy with BUB1 inhibition in breast cancer cell lines with functional ATM activity^36^. Therefore, cancer-specific DNA repair efficacy status should be taken into account for these compounds, also supported by the role of p53 in the response to DNA damaging anti-cancer agents^63^.

Our results included topoisomerase inhibitors topotecan, etoposide and doxorubicin (TOP2A) and irinotecan (TOP1), showing sensitivity in BRCA, OV, and STAD for TOP2A inhibitors, and resistance in COAD for the latter, at high BUB1 levels. Inhibition of BUB1 resulted in an antagonistic effect with topoisomerase inhibitor irinotecan^36^, while etoposide-driven apoptosis has been shown to be dependent on cleavage of BUB1 in vitro^64^, suggesting that high BUB1 levels may help in achieving the apoptotic effect. Functional SAC and error-free mitosis have been long known to be essential for the efficacy of topoisomerase inhibitors^56,65^, and BUB1 plays a crucial role for the recruitment of topoisomerase 2A via histone H2A phosphorylation^66^. We also report novel associations of high BUB1 with sensitivity to histone deacetylatase (HDAC) inhibitors (e.g. vorinostat and entinostat) through cell line drug screens, which could be linked to BUB1 by the interference of HDAC inhibitors with the SAC^67^.

Among drug hits identified through in vitro screens data, we also spotted Aurora kinase and Cdk1 inhibitors, which halt cell cycle progression of tumour cells at the checkpoint, resulting in G2/M phase cell cycle arrest. We identified previously unreported associations with small molecules such as alisertib (Aurora A inhibitor), AZD7762 (Chk inhibitor), BI-2536 (PLK1 inhibitor), and bortezomib (proteasome inhibitor), all of which participate in the function of the SAC alongside BUB1^68,69^. Previous studies have shown that inhibition of Aurora A sensitises glioblastoma cells to the alkylating agent temozolomide^70^, highlighting the potential for combinatorial treatments. We also uncovered several novel BUB1-drug associations, and particularly compounds that are not restricted to its mitotic role. An interesting association were cellular receptors and MEK/ERK target drugs, including crizotinib and selumetinib re-occurring in most analyses, which are involved in signalling pathways^71^. In addition to its role in mitosis, BUB1 participates in the regulation of receptor-mediated signalling cascades^72,73^. For instance, BUB1 was found to be crucial in regulating TGFβ signalling by promoting the TGF receptor complexes that are required for the induction of down-stream effectors (SMAD, RAS/MAPK, and PI3K/AKT)^74^.

We describe previously unreported associations of BUB1 with anti-angiogenic drugs targeting VEGF/R and EGFR (e.g. cetuximab, bevacizumab, sorafenib) and several tyrosine kinase inhibitors. In fact, BUB1 has also been shown to participate in membrane-mediated signalling and regulation of EGFR via endocytosis, with BUB1 inhibition reducing EGF signalling^75^. High BUB1 expression has been associated with promotion of hypoxia-mediated stemness in lung cancer^76,77^, which is tightly linked to tumour-sustaining angiogenesis, in line with our results showing hypoxia and stemness scores as highly correlated with BUB1 expression in the majority of cancers analysed. Guo et al.^77^ identified BUB1 among a set of core genes involved in the regulation of hypoxia and tumour stemness, including CENPF, BUB1B, KIF, and TTK, which we also found as top differentially expressed genes in BUB1 high vs BUB1 low patient cohorts. Indeed, most cancers and in vitro analyses showed sensitivity to EGFR/VEGFR targeting drugs, coherent with BUB1’s role in regulating membrane signalling^72–75^.

Overall, our results indicate that BUB1 expression can affect the mechanism of different drugs at various points in the pathway axis, from its functions in cell division to its modulation of cellular signalling. Several drug associations in the clinical analysis of the TCGA cohort showed cancer-specific patterns of sensitivity/resistance, which may be attributable to the heterogeneity of patient data, as well as differences in clinical and phenotypic features associated with BUB1. BUB1 was found to be overexpressed in the majority of cancers of the TCGA dataset, also highlighting a potential use as a diagnostic tool. Dysregulation of BUB1 has been described in a variety of tumours, including T-cell leukaemia, adenoid cystic carcinoma, bladder cancer, liver cancer, and breast cancer^19,20,23,34,35,74^. In agreement with our findings, BUB1 is associated with proliferation in cancer cells lines^78–80^ and depletion of BUB1 can reduce migration and invasion behaviour of cancer cells of the lung^74^, the breast^17^, and the liver^80,81^. In fact, BUB1 expression has already been reported as a prognostic marker, with high expression levels correlating with worse survival rates in breast^17^, liver^80,81^, bladder^30^, and neuroblastoma^82^. Nevertheless, variability in expression patterns is known in the literature, including subtype-specific up- or downregulation of BUB1 compared to normal tissue^24,26–28,83–85^. In our analysis, we found TGCT to be the only tumour exhibiting BUB1 downregulation; while BUB1 was not assessed specifically, expression of spindle genes was shown to correlate with TGCT histological subtypes, as observed by differential expression patterns (up- or downregulation) in in situ carcinomas, seminomas or non-seminomas^86^. Notably, we also identified a significant association of BUB1 with aggressive molecular subtypes and clinical features in different cancers, as well as phenotypic markers (i.e. hypoxia, stemness, and proliferation). These features, which are known to mediate resistance to anti-cancer drugs^87–89^, may play a role in the cancer-specificity of drug associations with BUB1 expression, particularly for those drugs that are not associated with mitotic machinery directly; detailed analysis of BUB1 expression in different cancer subtypes will inform disease biology and appropriate treatment strategies.

Taken together, we compiled potential compounds that are influenced by BUB1 expression. In addition to the drugs identified in the TCGA cohort, we screened publicly available repositories of drug responses and gene expression profiles. While the validation of these associations warrants experimental proof, and will likely be cancer-specific, these predictions can serve as a pre-screen for future experimental designs. Drug sensitivity screens based on gene expression profiles from cell lines and clinical datasets have in fact produced promising results in in vitro and in vivo validations^90,91^. We foresee that BUB1 expression could be exploited to either better inform treatment choices based on response prediction, or to modulate its expression (e.g. via an inhibitor) to alter the response of a given drug. The latter has the potential to solve issues related to treatment resistance and improve patient stratification, which continues to be a tedious clinical challenge in cancer care.

## Conclusions

In conclusion, we found diagnostic and prognostic applications for BUB1 expression in cancer, and predicted drugs that are associated with differential responses based on BUB1 levels. We foresee that BUB1 may be used as a therapeutic response marker to choose alternative treatments (including drug repurposing), as well as a pharmacological intervention to modulate BUB1 expression to improve drug response.

### Supplementary Information

Supplementary Information 1.

Supplementary Information 2.

Supplementary Information 3.

Supplementary Information 4.

Supplementary Information 5.

## Data Availability

Expression and clinical data used for this study are available from the TCGA-TARGET-GTEx cohort in the University of California Santa Cruz public repository Xena (https://xenabrowser.net), cBioPortal TCGA PanCancerAtlas (https://www.cbioportal.org), Genomic Data Commons Data Portal (https://portal.gdc.cancer.gov/). Cell line drug sensitivity data from the Genomics of Drug Sensitivity in Cancer (GDSC) project were downloaded from GDSC1000 resources (https://www.cancerrxgene.org/gdsc1000/GDSC1000_WebResources/Home.html); data from the Cancer Therapeutics Response Portal (CTRP) v2 was retrieved from the NCI CTD2 Data Portal (https://ctd2-data.nci.nih.gov/Public/Broad/).
