# Supplementary material for: Expression of the checkpoint kinase BUB1 is a predictor of response to cancer therapies
Source: Sci Rep. 2024 Feb 23;14:4461. doi: 10.1038/s41598-024-55080-y (PMC10891059; doi:10.1038/s41598-024-55080-y)
Supplement: Supplementary file 1 — Supplementary Information 1. [file 41598_2024_55080_MOESM1_ESM.docx]

**Supplementary Materials**

**Expression of the checkpoint kinase BUB1 is a predictor of response to cancer therapies**

Ylenia Cicirò, Denise Ragusa and Arturo Sala


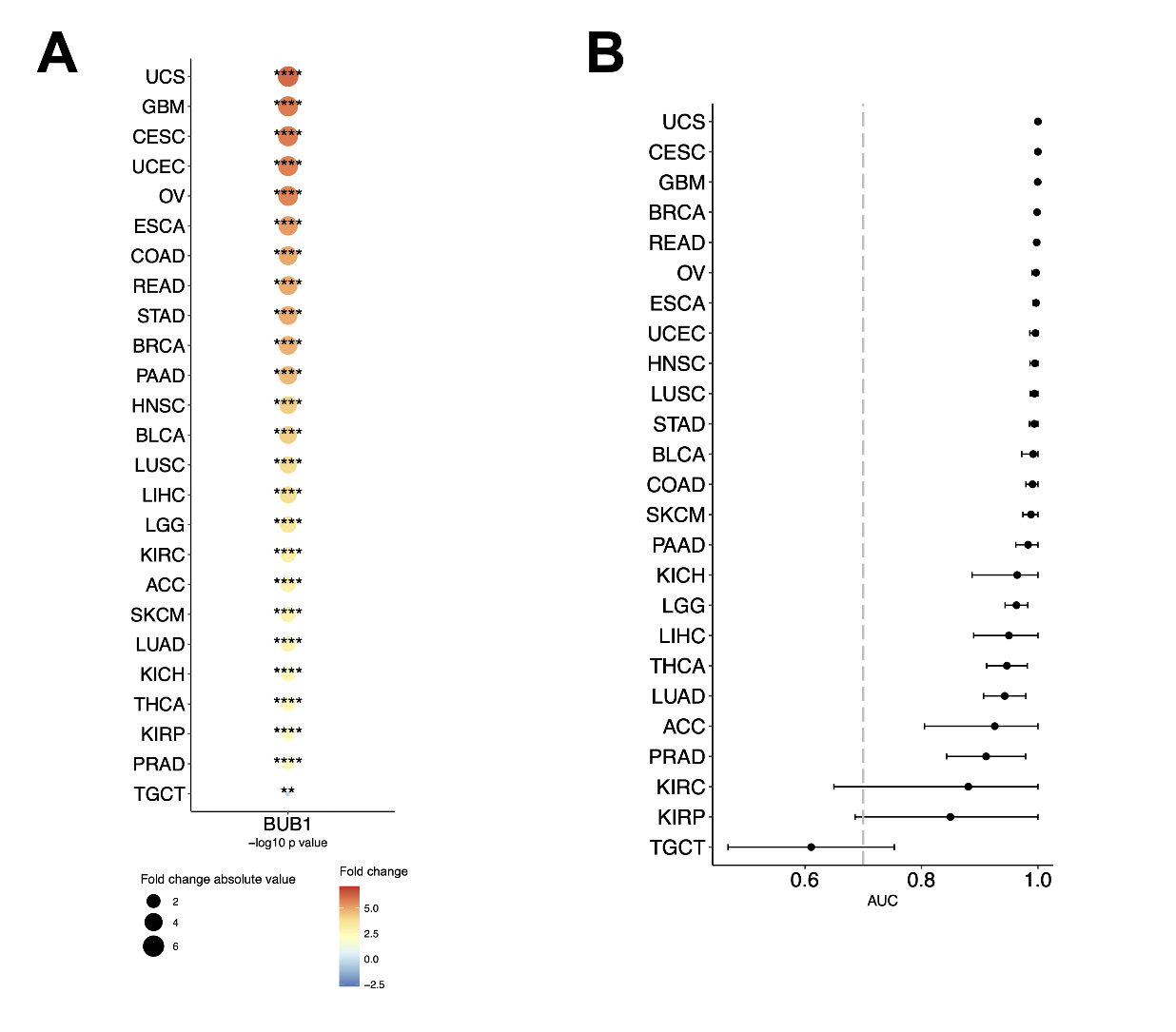


**Supplementary Figure 1 -** Expression of BUB1 in different cancers of the TCGA project. **A)** Expression fold change of BUB1 between tumour and normal tissues. Size of the dots represents the absolute value of fold change. Colours indicates the fold change. p value ≤ 0.05 (*), 0.001 (**), 0.0001 (***), and 0.00001 (****). **B)** Area under the curve (AUC) values from receiver operating characteristic (ROC) analysis to determine sensitivity and specificity of BUB1 expression in distinguishing tumour vs normal samples. Error bars indicate 95% confidence interval (CI).
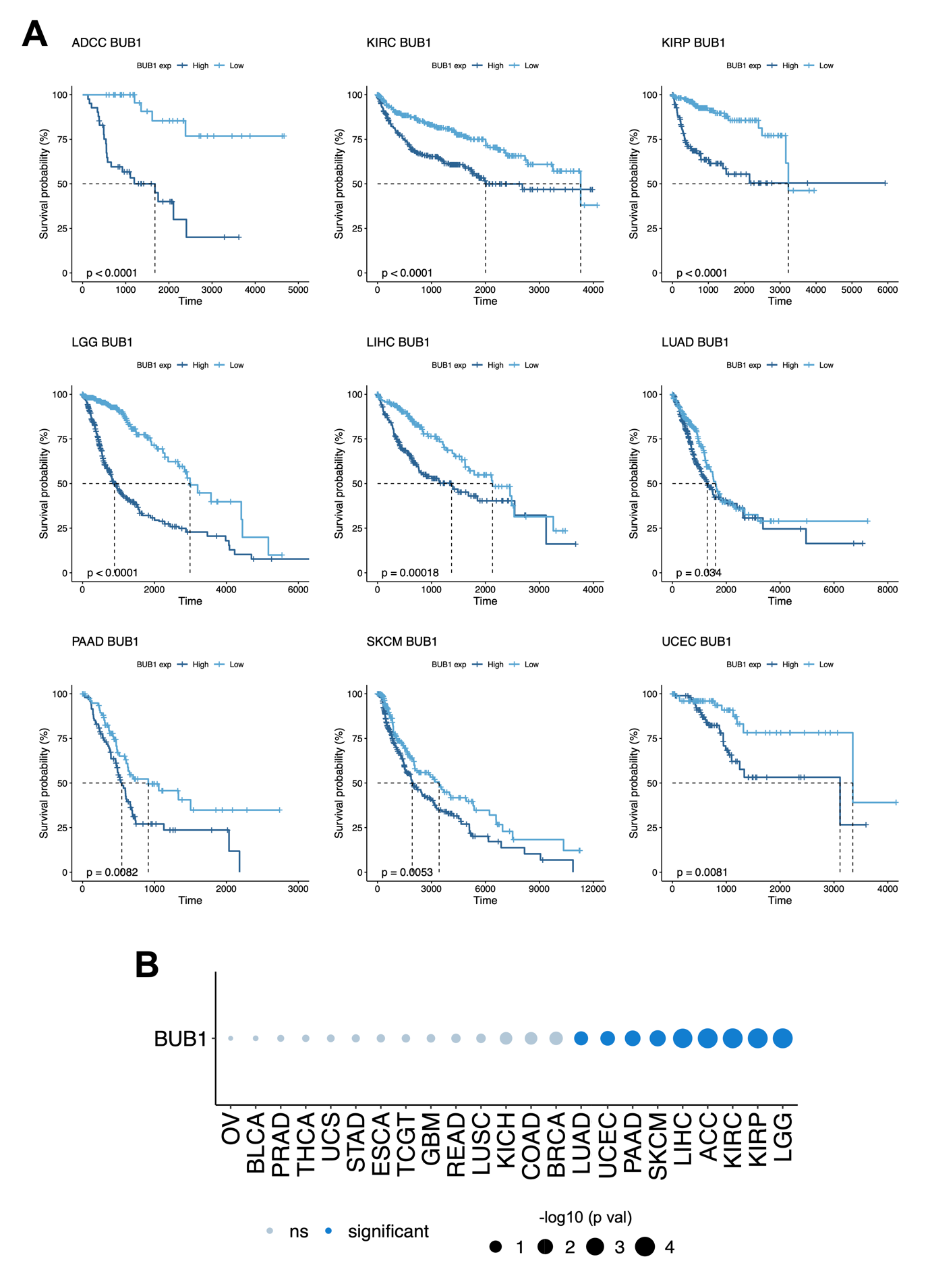


Supplementary Figure 2 – Survival analysis of BUB1 expression. A) Statistically significant Kaplan-Meier plots of overall survival (OS) according to BUB1 expression levels defined as high-low by mean cut-offs. B) Summary of Kaplan-Meier results on OS by BUB1 levels in all cancers analysed.


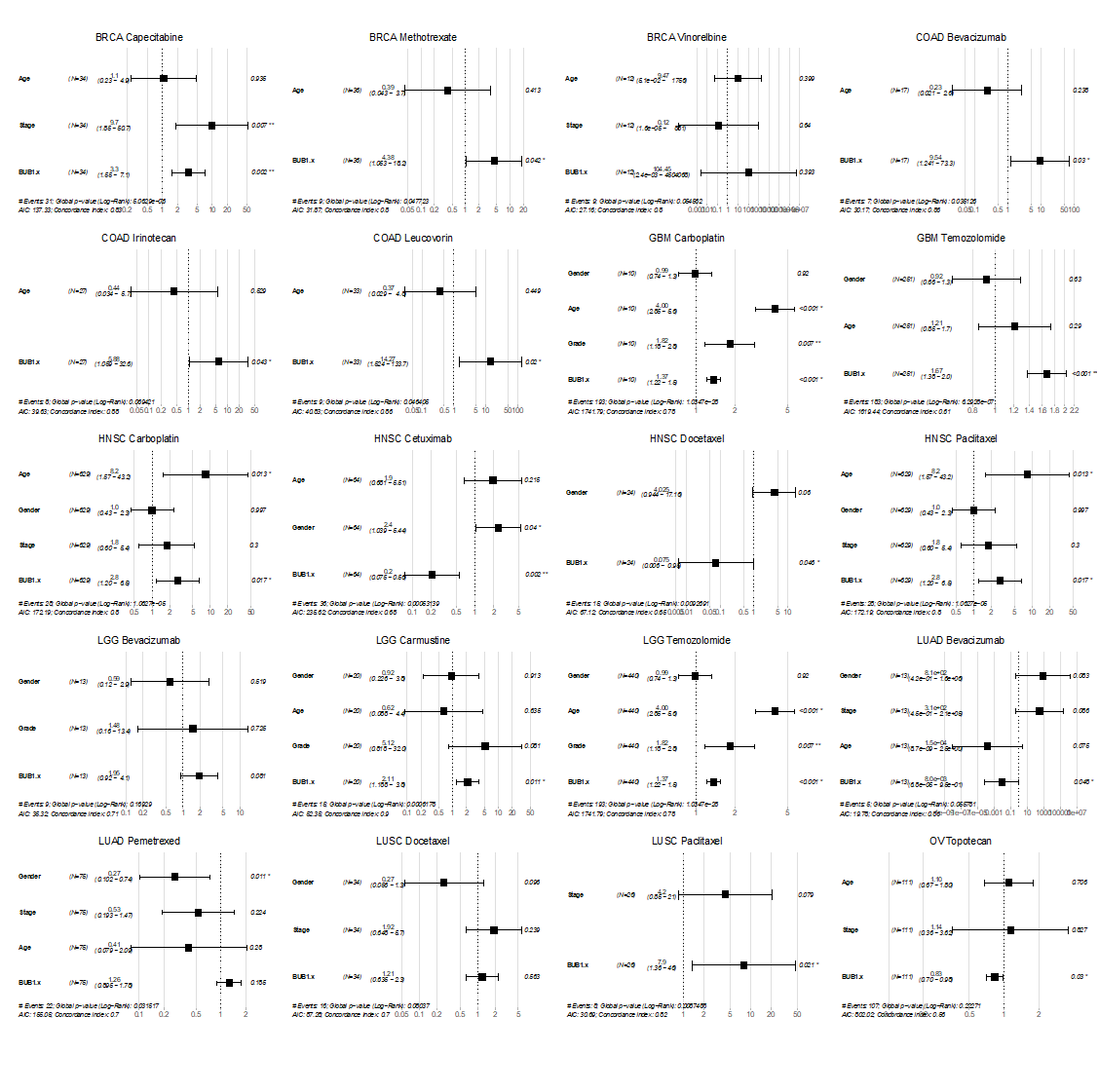


Supplementary Figure 3 – Multi-variate analysis on overall survival (OS) based on BUB1 expression and treatment type for each cancer.


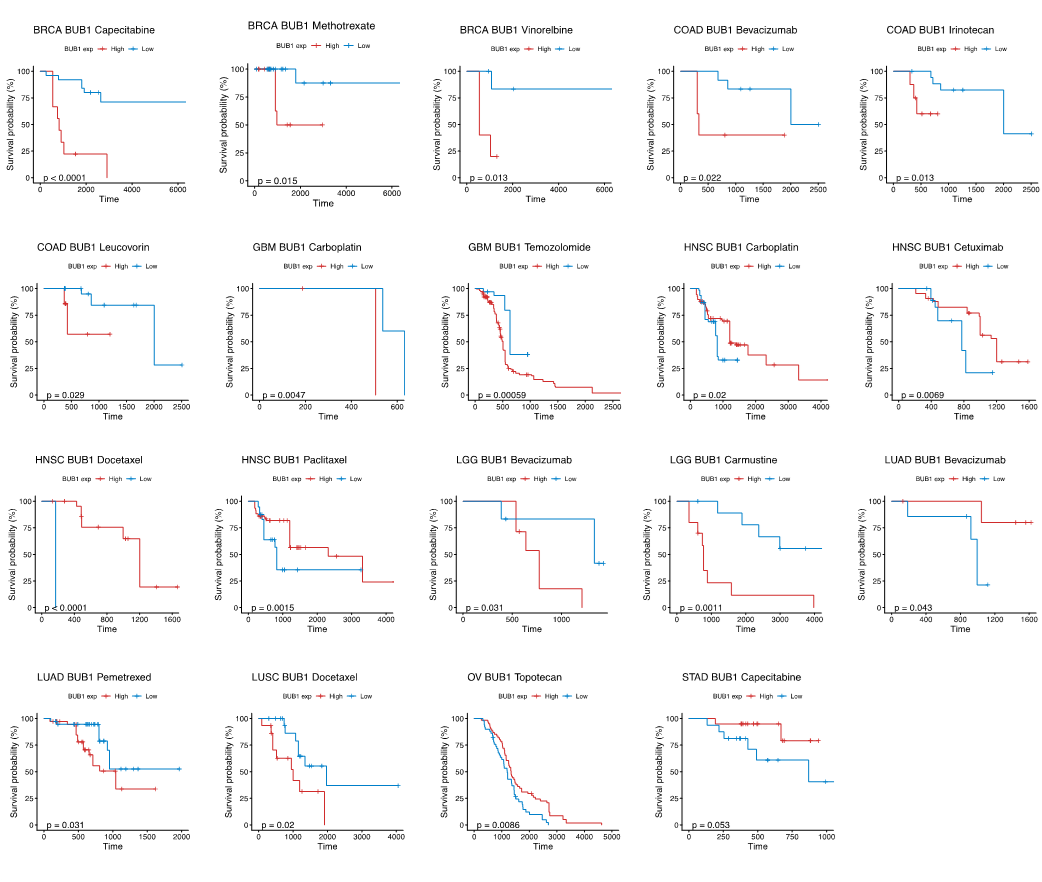


Supplementary Figure 4 – Kaplan-Meier plots of significant associations in overall survival by BUB1 expression and drug treatment.


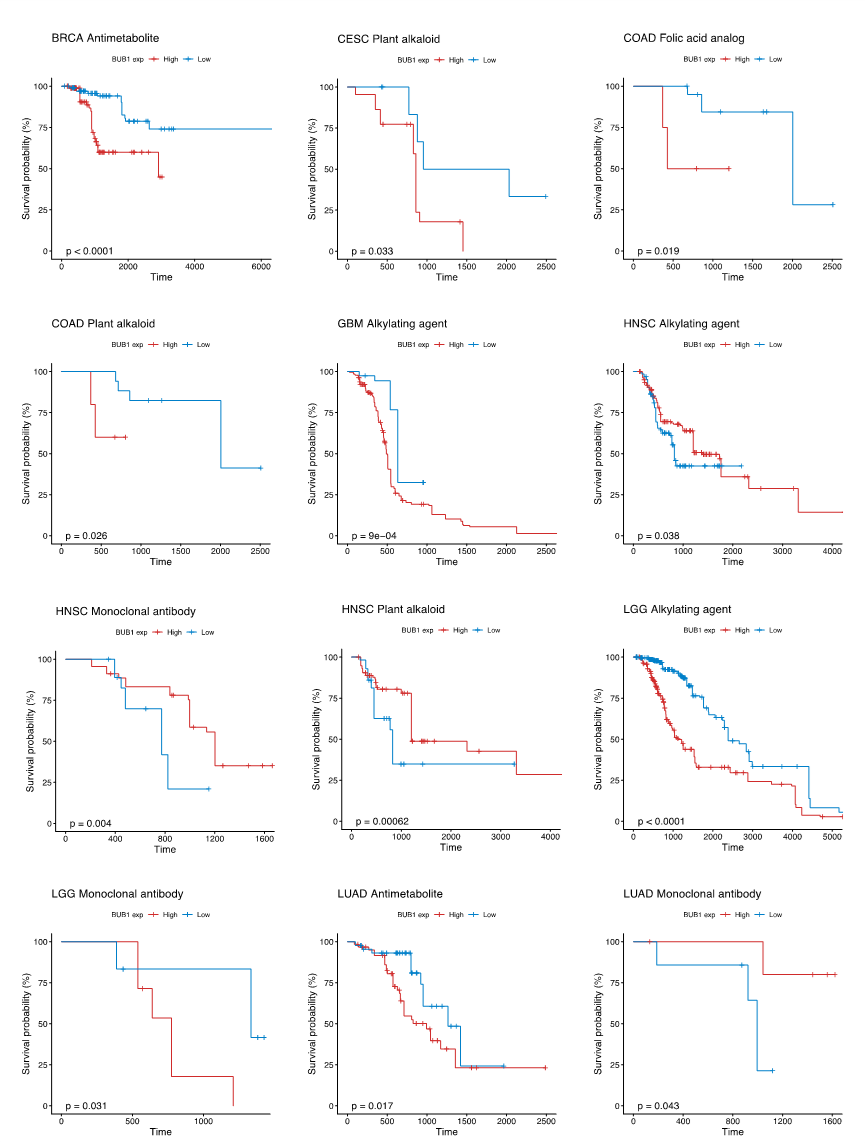


Supplementary Figure 5 – Kaplan-Meier plots of significant associations in overall survival by BUB1 expression and drug class.

**
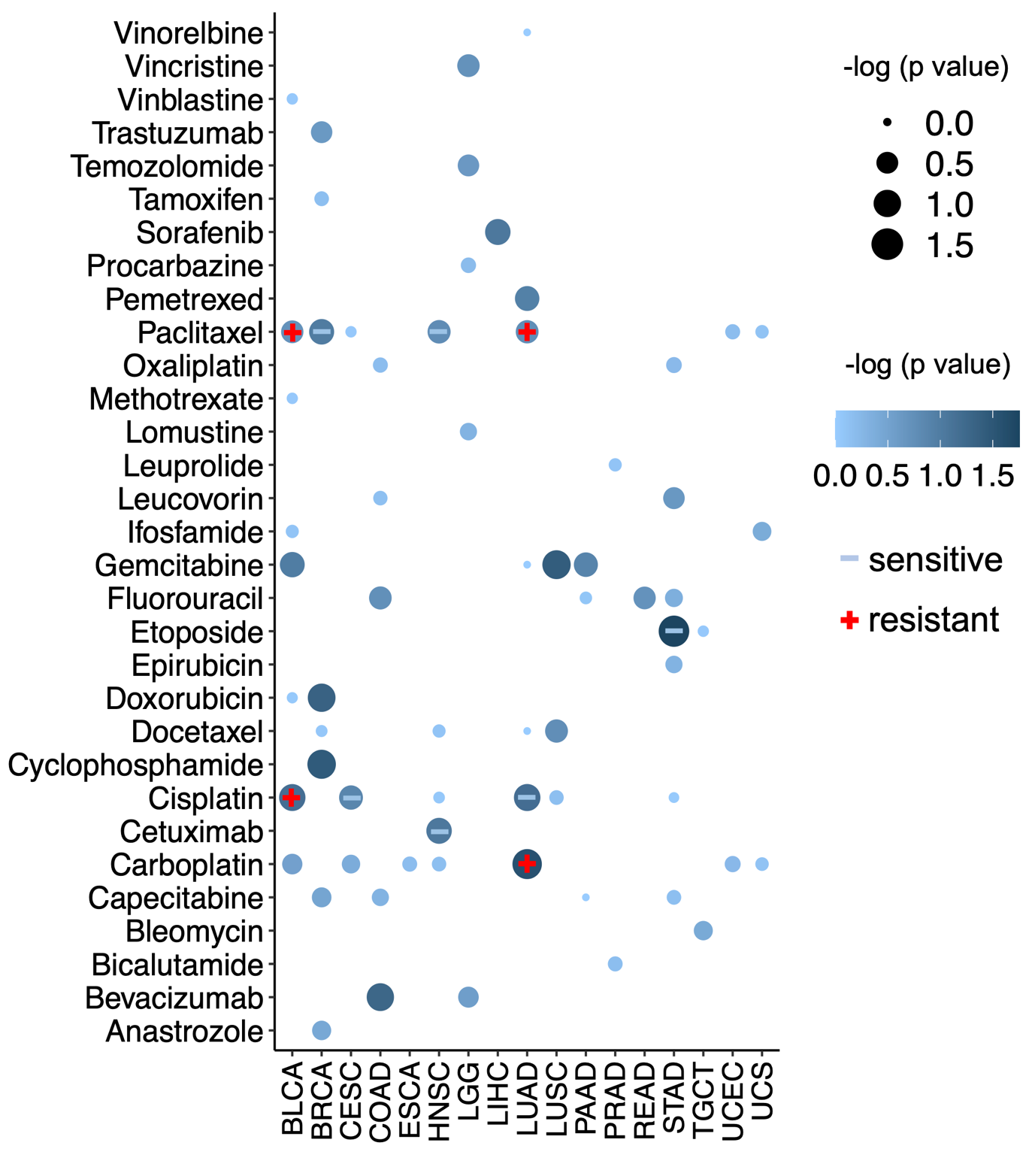
**

Supplementary Figure 6 – Summary of results of drug response data according to the RECIST classification system of disease progression according to BUB1 expression levels (high-low) by drug administered. The dots indicate the significance in -log 10 (p value) of the difference in mean expression values between BUB1 high and BUB1 low groups. Associations were classified as “resistant” (+) when BUB1 expression was higher in the non-responsive group, or “sensitive” (-) if BUB1 was higher in the responsive class.


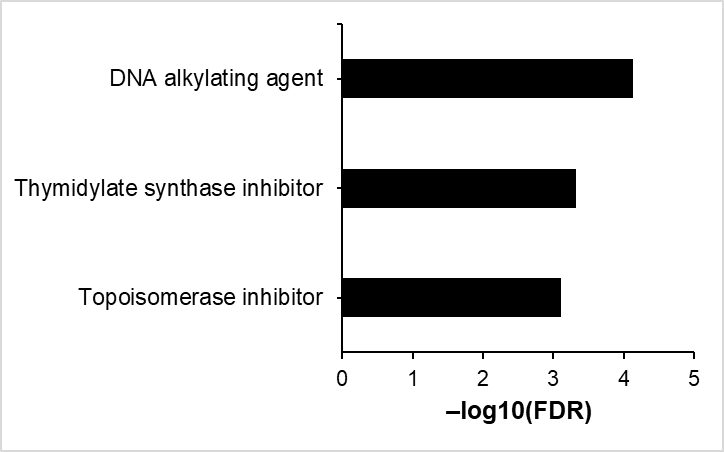


Supplementary Figure 7 – Drug Set Enrichment of mechanism of action of 20 drugs extracted from TCGA clinical data on BUB1 expression.


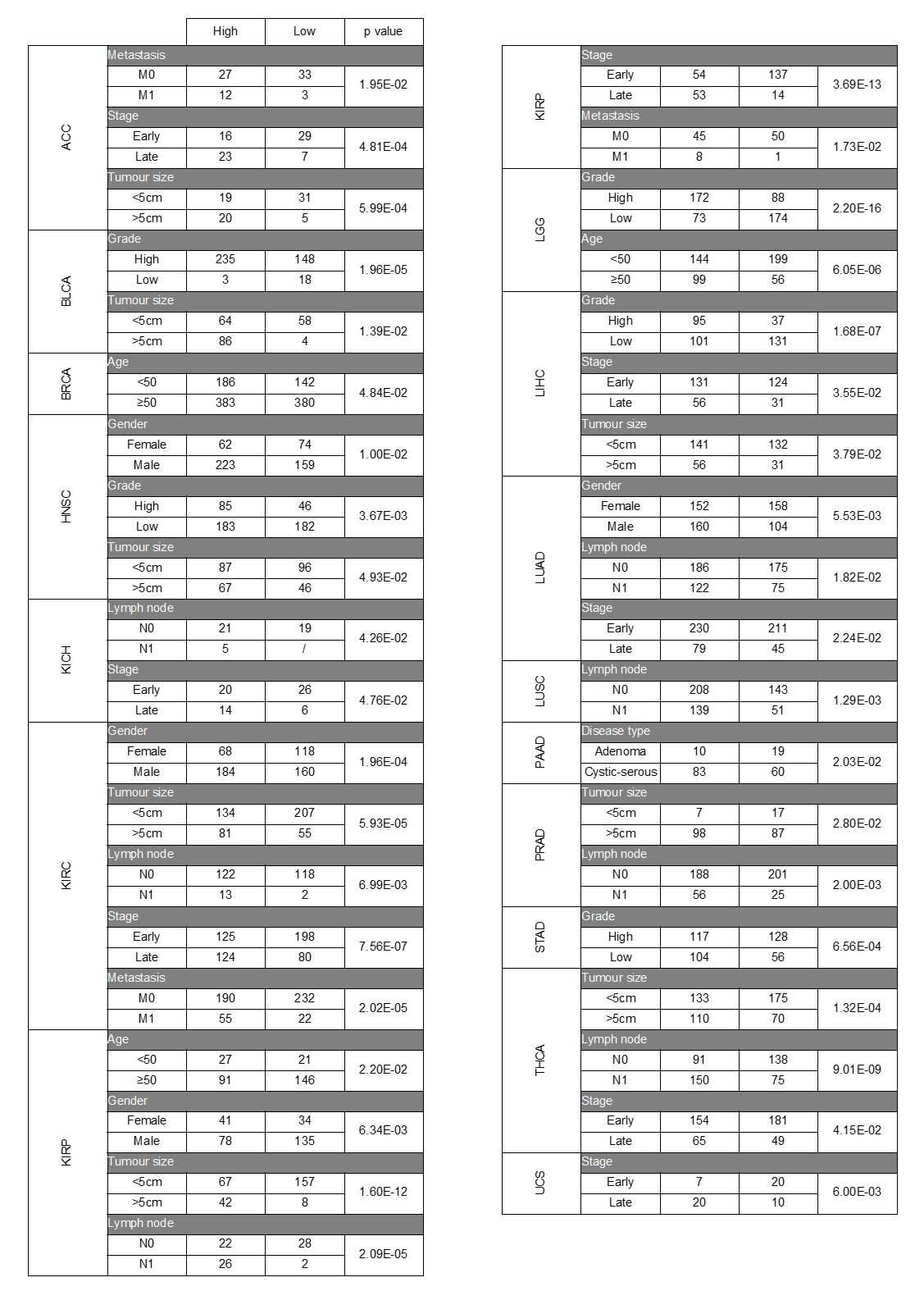


Supplementary Table 1 – Chi-square analysis of clinico-pathological features according to BUB1 levels.
